# Two-dimensional strain ultrasound speckle tracking as a novel approach for the evaluation of right hemidiaphragmatic longitudinal deformation

**DOI:** 10.3892/etm.2013.1133

**Published:** 2013-05-30

**Authors:** XIONG YE, HUI XIAO, WEIMING BAI, YONGJIE LIANG, MING CHEN, SUIYANG ZHANG

**Affiliations:** 1 Medical College of Soochow University, Suzhou, Jiangsu 215123;; 2 Department of Laboratory Medicine, Shanghai First People’s Hospital, Shanghai Jiaotong University, Shanghai 200080;; 3 Departments of Ultrasound, East Hospital, Tongji University, Shanghai 200120;; 4 Respiratory Medicine, East Hospital, Tongji University, Shanghai 200120;; 5 Department of Respiratory Medicine, The Second Artillery General Hospital, Beijing 100088, P.R. China

**Keywords:** diaphragm kinetics, ultrasound, M-mode, speckle tracking, strain

## Abstract

The diaphragm is an important respiratory organ. The aim of this study was to quantitatively evaluate the longitudinal deformation of the right hemidiaphragm in normal subjects using two-dimensional strain ultrasound speckle tracking. Twenty-one healthy subjects were enrolled in this study. GE Healthcare Vivid E9 equipment with M5S probe and Q-analysis software were used. Negative strain values first appeared in the zone of apposition and then in the crura of the right hemidiaphragm in the inspiratory phase; the dome of the diaphragm was observed to be passively stretched. The longitudinal strain of the right hemidiaphragm in the zone of apposition was higher than that in the crura in forced breathing (P=0.024). The strains of the whole diaphragm and the zone of apposition changed significantly in quiet (P=0.000) and forced breathing (P=0.005). Ultrasound strain imaging may quantitatively assess diaphragm deformation and provide another useful modality for evaluating diaphragm kinetics.

## Introduction

The diaphragm is a dome-shaped sheet of muscle with a central tendinous component that separates the thorax from the abdomen and maintains the pressure gradient between those two cavities (1). It arises from the crura, the arcuate ligaments, the costal margin and the posterior aspects of the xiphoid (2). It has essential anatomic and functional roles necessary for the breathing process, which undertakes 60–75% of the total tidal volume of respiration (3).

The morphology and movement of the diaphragm may be detected by fluoroscopy, chest radiography, computed tomography (CT) and magnetic resonance imaging (MRI) (4–7). Using these techniques to diagnose weakness or paralysis of the diaphragm results in exposure to radiation, requires patient transportation and is time-consuming. Ultrasound (US) allows direct visualization of the displacement of the diaphragm in humans breathing freely, as well as inspection of critical patients at the bedside without the use of ionizing radiation. Several authors have reported the use of M-mode US for the evaluation of the diaphragmatic displacement (8–10); however, their results were inconsistent. Different positions of the patients, the transducer placement, the direction of the US beam and the compressive maneuvers during US examination may affect the results of the measurements (11). Furthermore, M-mode US measurements only reflect the diaphragmatic motion where the exploration line is. Thus, different locations of the exploration line generate different results, due to the asynchronicity of the diaphragmatic motions.

In recent years, 2-dimensional (2D) deformation imaging (strain and strain-rate imaging) has emerged as a new noninvasive method for assessing myocardial function and differentiating between active and passive movement of myocardial segments (12). It analyzes the myocardial motion by tracking speckles (natural acoustic markers) in the 2D US image. The geometric shift of each speckle represents local tissue movement (13). In this study, we describe a novel method utilizing 2D strain imaging to quantify segmental longitudinal deformation (strain) of the right diaphragm using specially designed software for tracking cardiac motion.

## Materials and methods

### Subjects, setting and study design

Twenty-one healthy volunteers (12 male and 9 female) without specific sporting training were selected from April 1, 2012 to June 30, 2012 in the Ultrasound Department, Shanghai East Hospital affiliated to Tongji University. None of the subjects had a history of diaphragm dysfunction, chronic obstructive pulmonary diseases, asthma, thoracic surgery, pleural thickening or smoking. No fasting or preparation was required. After providing informed consent, all subjects underwent a medical history interview, physical examination and pulmonary function tests by standard spirometry (Ilmeter 1304; Masterlab Jaeger, Würzberg, Germany) according to the standards of the American Thoracic Society. The criteria for classification as normal consisted of a forced vital capacity (FVC) >80% of predicted, a forced expiratory volume in 1 sec (FEV1) >80% of predicted and a FEV1/FVC ratio >80% of predicted. The measurements of right hemidiaphragm kinetics were made on the B- and M-mode frozen images using the US machine calibration and algorithm in supine position during quiet and forced breathing. The study was performed following the approval of the ethics committee of Shanghai East Hospital affiliated to Tongji University.

### US technique

**Figure 1.**
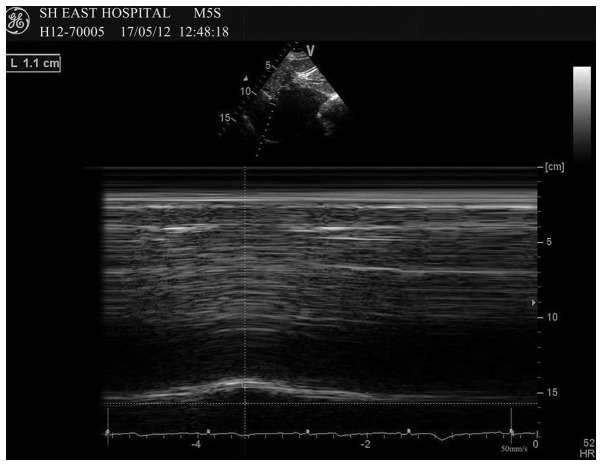
M-mode US of quiet breathing and corresponding cardiac cycle under the hyper-echogenic curve in a healthy subject. US, ultrasound.

**Figure 2.**
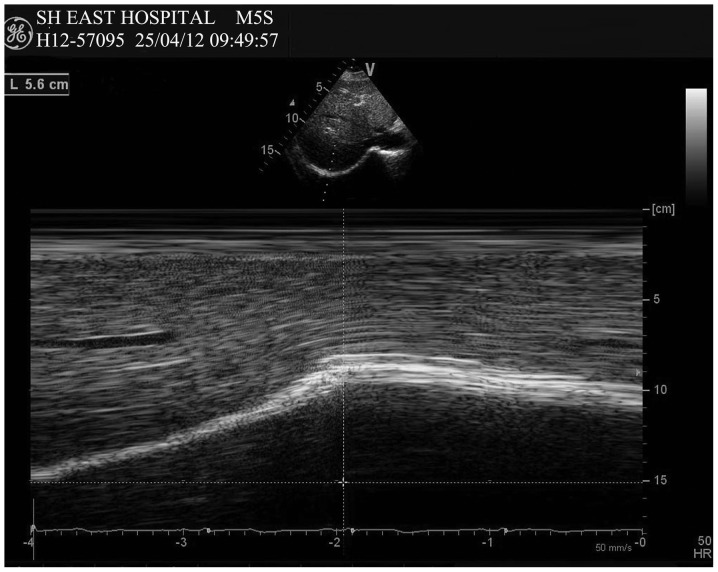
M-mode US of forced breathing and corresponding cardiac cycle under the hyper-echogenic curve in a healthy subject. US, ultrasound.

US examinations were performed by an experienced investigator using a commercially available Doppler echocardiograph (Vivid E9 Diagnostic Ultrasound System; GE Healthcare, Horten, Norway), with an appropriate total gain and depth, 50–70 frames/sec and equipped with a M5S convex transducer. Assessment of right diaphragm excursion on the cranial-caudal axis was performed in B- and M-mode, with a frequency between 3.5 and 5.0 MHz depending on the depth of the structure for optimal visualization. The excursion of the right hemidiaphragm was measured from the condition of functional residual capacity to reaching tidal volume (quiet breathing; Fig. 1) and total lung capacity (forced breathing; Fig. 2). To obtain the images, the transducer was positioned on the abdominal wall just below the right costal margin around the midclavicular line with the right intrahepatic vein branch as an anatomical landmark. In this view, the right hemidiaphragm appeared as a thick hyper-echogenic curved line. The transducer was firmly held in this position during all phases of the respiratory cycle. M-mode US of right diaphragm motions and electrocardiogram were performed synchronously with a 50 mm/sec paper speed.

### Diaphragm strain measurements

**Figure 3.**
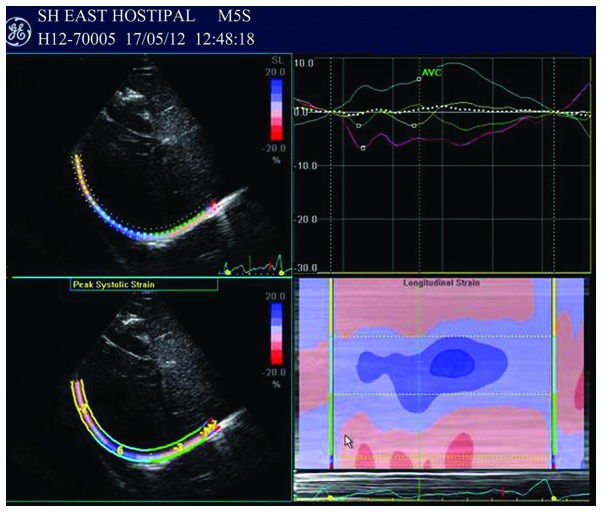
Strain values of the second cardiac cycle in Fig. 1. Longitudinal whole strain (dotted white curve) and regional longitudinal strain curves (distinctively colored curves for three diaphragmatic segments) obtained from intrahepatic vein branch views by 2D strain imaging in a healthy subject. The analysis software was specially designed for tracking myocardial segmental strain. It divides the ROI of the myocardium into six segments medially. The red curve in the right upper quadrant includes three segments. In this study, the value of the red curve was abandoned and the non-overlapping curve (yellow, blue and green) was used to represent the diaphragm strain. ROI, region of interest.

**Figure 4.**
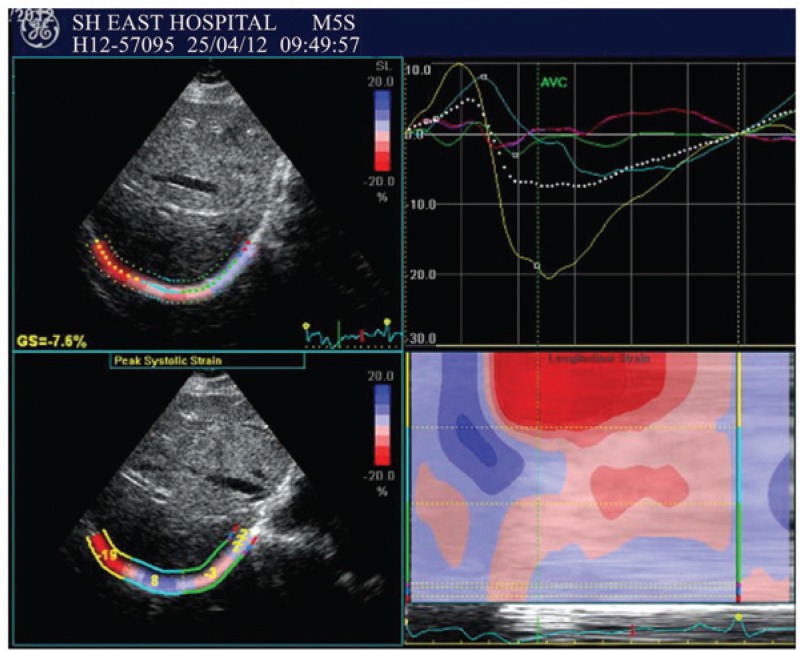
Strain values of the second cardiac cycle in Fig. 2. Longitudinal whole strain (dotted white curve) and regional longitudinal strain curves (distinctively colored curves for three diaphragmatic segments) obtained from intrahepatic vein branch views by 2D strain imaging in a healthy subject. The red curve in the right upper quadrant includes three segments. In this study, the value of the red curve was abandoned and the non-overlapping curve (yellow, blue and green) was used to represent the diaphragm strain.

The videos of the right diaphragm motions for four continuous cardiac cycles were saved and diaphragm strains were detected using automatic function imaging and cardiac cycle analysis in M-mode US in turns. The peritoneum, mid-diaphragm and pleural border were equivalent to the epicardial, midmyocardial and endocardial lines according to the manual of the analytical software devised for cardiac motion as a region of interest (ROI). After stopping the motion of the cursor for several seconds, the software detected longitudinal deformation of the diaphragm and exhibited the results automatically. The value obtained from the cardiac cycle under the maximal inspiratory slope represented the longitudinal deformation of the diaphragm in the inspiratory phase (Figs. 3 and 4). The tracking time was from the R wave to aortic valve closure (AVC) in the same cardiac cycle. The right diaphragm hyper-echogenic curved line was divided into three segments in the majority of the results: the dome position (the highest point of the diaphragm) in the middle; the zone of apposition (the cylindrical region of the diaphragm that apposes the lower rib cage) in the right; and the crural of the diaphragm in the left. A negative value indicated active systole of the diaphragm-corresponding segment and a positive value indicated passive stretching.

### Statistical analysis

Statistical analysis of data was performed using SPSS 16.0 software (SPSS, Inc., Chicago, IL, USA). Continuous variables are expressed as mean ± standard deviation. The distribution of data was analyzed with a Kolmogorov-Smirnov test. For a normal distribution, differences were compared using an unpaired Student’s t-test and data that were not normally distributed were compared using the Mann-Whitney U test. P<0.05 was considered to indicate a statistically significant difference.

## Results

**Figure 5.**
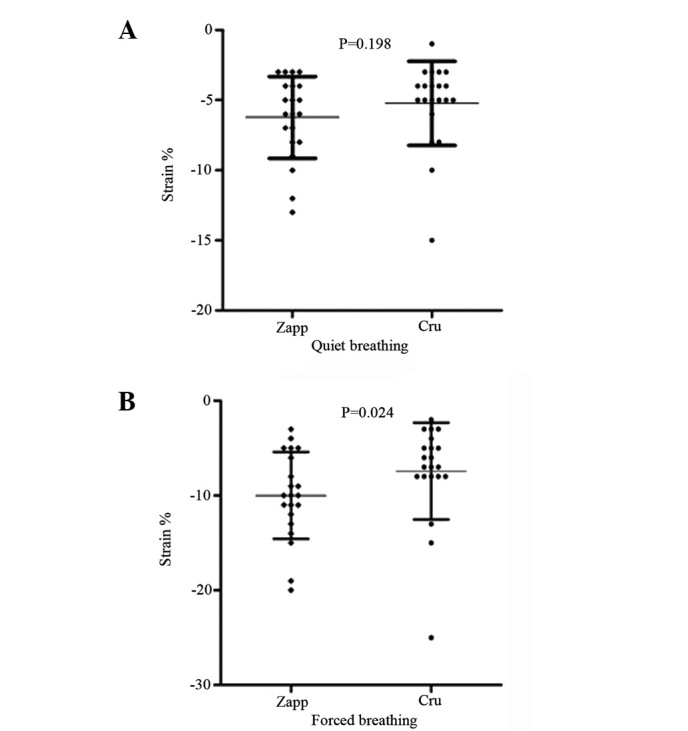
Comparison the longitudinal strains of the zone of apposition (Zapp) with the crura of the diaphragm (Cru) in quiet and forced breathing.

Twenty-one healthy volunteers were enrolled in the present study. Demographic, anthropometric and spirometric data of the volunteers are reported in Table I. Pulmonary function assessments by spirometry were normal in all subjects. The mean quiet and forced diaphragm excursion values were 15.52±0.60 and 59.29±1.88 mm, respectively, measured by M-mode US. Negative strain values first appeared in the zone of apposition and then in the crura of the right diaphragm, through analysis the cardiac cycle under M-mode US. The absolute value of the negative strain values increased from the beginning of inspiration to the maximal inspiratory slope. The right diaphragm real-time 2D segmental strains are reported in Table II. Positive strain values describe passive extension and negative values describe active shortening of a given diaphragm segment related to the length at a previous time point. In quiet breathing, there was no significant difference in the strains of the crura of the right diaphragm and the zone of apposition (P=0.198); however, there was a significant difference in forced breathing (P=0.024; Fig. 5).

## Discussion

The diaphragm is active throughout the life of an individual. Dysfunction of the diaphragm, including paralysis, weakness and eventration, is a frequent contributor to dyspnea. Despite its importance, the diaphragm is often underappreciated and incompletely evaluated by clinicians and radiologists (14). M-mode US allows visualization of the displacement of the diaphragm. The role of M-mode US in the qualitative assessment of diaphragm amplitude has been investigated in normal and pathological conditions (15–17). Our results of M-mode US measurements are similar to these previous findings. Motion measurements do not differentiate between active and passive movement of a moving object, whereas deformation analyses (strain imaging) allow discrimination between active and passive tissue movement (12). Strain imaging (deformation analysis) is more useful than wall motion analysis (velocity and displacement) for the detection of regional myocardial dysfunction (18). Longitudinal strain of the diaphragm may be as important to its function as myocardial strain is for cardiac function.

In this study, 2D strain US speckle tracking was used as a novel approach for analyzing right hemidiaphragm deformation in healthy subjects. After analyzing the cardiac cycle in each individual using M-mode US, we found that negative strain values first appeared in the zone of apposition and then in the crura. The negative strain values describe active shortening of a given segment of the diaphragm related to the length at a previous time point. The positive strain values describe passive extension; the dome of the diaphragm stretches passively. The right diaphragm longitudinal strains of the crura and the zone of apposition presented no significant difference in quiet breathing; however, there was a significant difference in forced breathing (P=0.198 and P=0.024, respectively). The strains of the whole diaphragm and the zone of apposition changed significantly in quiet and forced breathing (P=0.000 and P=0.005, respectively); however, there were no differences in the crura and the dome (P=0.071 and P=0.278, respectively). The possible interpretation is that the costal and crural segments of the diaphragm have different embryological origins, different segmental innervations and different functional attributes (2). There are regional differences in diaphragm thickness, *in vivo* fiber length and the degree of shortening within the costal diaphragm of anesthetized dogs during passive lung inflation (19). This suggests that the potential of generating force and causing displacement is not uniform throughout the diaphragm (19). However, Suzuki *et al* examined the shortening of the parasternal intercostal muscles, crural diaphragm and costal diaphragm in dogs through implanted sonomicrometers. The authors identified no difference in the shortening pattern between crural and costal diaphragms (20). The advantage of right diaphragm strain imaging using non-Doppler 2D speckle tracking is that it tracks in two dimensions, along the direction of the longitudinal shortening and extension of the diaphragm, not along the US beam, and since it is not based on tissue Doppler measurements, it is angle-independent (18). The non-Doppler 2D speckle tracking provides a bedside, non-invasive and low-cost examination method for detecting changes of diaphragm length, compared with dynamic CT and MRI.

There is a limitation that the analysis time is from the R wave to AVC in one cardiac cycle, which corresponds to the maximum inspiratory slope under M-mode US, and the results only reflect diaphragm deformation of this short time period. These results do not represent the maximum deformation of the diaphragm in an entire inspiratory phase. One potential solution for this limitation is to develop new specialized software that is triggered by the breathing cycle to allow for a simple, fast, accurate and reproducible measurement of diaphragm deformation. Another limitation of this study is that it was performed on only 21 healthy volunteers without grouping according to age and gender. Although it is difficult to draw major conclusions on such small numbers, we consider that based on these preliminary results, 2D strain US speckle tracking has the potential to detect deformation of the diaphragm. All analyses were performed by one skilled US expert; therefore, the intra- and inter-observer variability is unknown. Further studies on diaphragm speckle tracking compared with sonomicrometer or MRI tagging as a reference are required to validate the precision of this technique for measurement of diaphragm mechanics.

In conclusion, we have demonstrated the potential application of 2D strain US speckle tracking in the evaluation of right diaphragm deformation, and shown that it is a promising new tool for the quantification of diaphragm function. The method is safe and may enhance our understanding and diagnosis of abnormal diaphragm mechanics in pulmonary disease. Further studies for tracking diaphragm strain in a larger number of patients with obstructive or restrictive lung disease, receiving mechanical ventilation or before and after thorax or abdominal surgery are required.

## Figures and Tables

**Table I.** Demographic data, anthropometric characteristics and pulmonary function spirometry test results of the healthy volunteers.

Characteristics	Value
Number of subjects	21
Gender (M/F)	12/9
Age (years)	43±11
BMI (kg/m^2^)	22±3.0
Heart rate	65±12
FVC (predicted %)	3.9±1.1
	98.2±11.0
FEV1 (predicted %)	3.7±1.6
	96±13.2
FEV1/FVC (predicted %)	89±7.5
	98.2±10.3

Values for age, BMI, heart rate, FVC, FEV1 and FEV1/FVC are mean ± standard deviation. BMI, body mass index; FVC, forced vital capacity; FEV1, forced expiratory volume in 1 sec.

**Table II.** Right diaphragm real-time 2D segmental strains of the healthy volunteers.

Segment	Quiet breathing	Forced breathing	P-value
Crura of diaphragm	−5.24±3.00	−7.42±5.10	0.0709
Dome of diaphragm	3.24±1.64	4.10±2.34	0.2780
Zone of apposition	−6.24±2.91	−10.00±4.58	0.0051
Whole diaphragm	−2.14±1.80	−4.62±2.56	0.0002

Data are mean ± standard deviation.
